# Tilted seat position for non-ambulant individuals with neurological and neuromuscular impairment: a systematic review

**DOI:** 10.1177/0269215507082338

**Published:** 2008

**Authors:** SM Michael, D Porter, TE Pountney

**Affiliations:** Medical Physics and Engineering, Leeds University Hospitals NHS Trust, Leeds; School of Health and Social Care, Oxford Brookes University, Oxford; Chailey Heritage Clinical Services, Southdowns Health NHS Trust, Sussex, UK

## Abstract

**Objective**: To determine the effects of tilt-in-space seating on outcomes for people with neurological or neuromuscular impairment who cannot walk.

**Data sources**: Search through electronic databases (MEDLINE, Embase, CINAHL, AMED). Discussions with researchers who are active in field.

**Review methods**: Selection criteria included interventional studies that investigated the effects of seat tilt on outcome or observational studies that identified outcomes for those who had used tilt-in-space seating in populations with neurological or neuromuscular impairments. Two reviewers independently selected trials for inclusion, assessed quality and extracted data.

**Results**: Nineteen studies were identified which fulfilled the selection criteria. Seventeen of these were essentially before–after studies investigating the immediate effects of tilting the seating. All studies looked at populations with neurological impairment, and most were on children with cerebral palsy (*n* = 8) or adults with spinal cord injury (*n* = 8).

**Reviewer**'**s conclusion**: Posterior tilt can reduce pressures at the interface under the pelvis.

## Introduction

Tilt-in-space wheelchairs and seats are increasingly used by people with neurological or neuromuscular impairments who cannot walk. Tilt-in-space systems may be considered for a variety of reasons, including low sitting tolerance or discomfort, a requirement to rest in the seat, and to assist with manual handling.^1^ Drawbacks to these systems compared with conventional wheelchairs and seats include purchase costs, size and complexity of equipment. Tilt-in-space wheelchairs are also heavier and less manoeuvrable than more standard wheelchairs due to a longer wheelbase, and this may restrict access to transport.^2^

A backwards-tilted sitting position has been suggested to improve head and trunk posture,^3^,^4^ and to reduce the loading under the buttocks^5–7^ or through the spine.^8^ There are concerns that seating that is excessively tilted back limits communication, upper limb function and the ability to stand up from the chair.^9^

A forward-tilted sitting position has also been proposed to maintain lumbar lordosis, decrease posterior pelvic tilt, reduce the effect of tight hamstrings on the position of the pelvis and to position a person within reach of the desk or table.^10^,^11^ Forward-tilted positions have been incorporated into some paediatric seating.^12^

With no evidence-based criteria or guidelines for provision and use of these systems, practices around the provision of tilt-in-space seating systems vary widely. Tilt-in-space seating may be provided by statutory service in some areas. Systems are also available for purchase directly by the user.

In a qualitative study^2^ of severely disabled wheelchair users with multiple sclerosis and significant spasticity themes such as wheelchair size and manoeuvrability, transport difficulties, comfort, pressure ulcers, sitting up during day for prolonged periods and fatigue emerged from in-depth interviews. Seven tilt-in-space and 16 conventional wheelchair users participated.

With this background it was thought that a systematic review on the effects of tilt-in-space seating might inform clinical practice on seating provision and use within these populations, and identify what further research studies on this topic are required in order to establish evidence-based guidelines for provision.

## Objective

To identify the effects of seat orientation on physiology; body parts and systems; and on activity for adults and children with neurological or neuromuscular impairments who cannot walk.

## Method

### Search strategy

A search was carried out in December 2006 of electronic databases including MEDLINE (1950–2006), Embase (1980–2006), CINAHL (1982–2006), AMED (1985–2006) using thesaurus terms ‘wheelchair’, ‘wheelchairs’, ‘seat’, ‘seating’ and free text words ‘tilt$’ and ‘tip$’ looking for articles in English on humans. Reference lists in studies and review articles were examined for other appropriate articles. A search for unpublished studies was conducted via contact with experts in the field.

### Selection criteria

**Figure 1:**
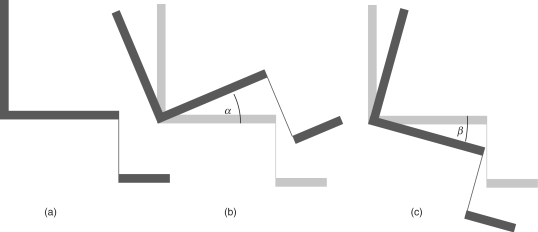
Schematic lateral views of seat showing (a) upright, (b) posteriorly and (c) anteriorly tilted seat orientations. α = posterior tilt angle, *β* = anterior tilt angle.

Studies were identified that investigated the effects of seat tilt on outcome for the seated individual. Experimental studies that compared outcomes at different angles of tilt were included as were observational studies that compared outcomes for those that had used tilt-in-space seating to those that had used a seat in a fixed orientation. A tilt of the seat was taken to be a rotation of the complete seat about a mediolateral axis, and tilt angle is as described in Figure 1.

Studies with both randomized and non-randomized allocation of subjects to seat or seat orientation were selected for review.

Studies included only participants who were non-ambulant and who had a congenital or acquired neurological or neuromuscular condition. Participants could be of any age.

Any outcome was considered that described the effects of seat tilt on physiology; body parts and systems; and on human activity including fulfilment of societal roles.

### Data collection and analysis

The two reviewers independently selected trials for inclusion, assessed quality and extracted data.

The methodological strength of each study was evaluated using a commonly used hierarchy of study designs from the NHS Centre for Reviews and Dissemination.^13^ Methodological strength was graded on a scale from 1 to 5 where 1 is the highest level (Table 1).

**Table 1:** Levels of evidence^13^

Level	
1	Experimental study (e.g. RCT with concealed allocation)
2	Quasi-experimental study (e.g. experimental study without randomization)
3	Controlled observational study: (a) cohort study, (b) case–control study
4	Observational study without control group
5	Expert opinion based on pathophysiology, bench research or consensus

Quality was also assessed in addition to methodological strength. This was based on: whether the study was properly controlled; what methods of randomization or allocation to intervention groups were used; and whether the groups were comparable at baseline. The roles of chance, confounding and bias in the study were also considered. Attempts were made to contact authors to obtain any important data that were missing and necessary for the review.

The studies included in this systematic review were not only randomized control trials. This is because studies have tended to focus on instantaneous outcomes as a result of being tilted compared with upright and alternative designs have often been used (e.g. cross-over trials). However, in appraising such studies particular attention was given to identifying potential sources of bias. Cross-over trials could be rated at levels 1, 2, 4 or 5 depending on samples size and homogeneity; whether the effects of order, timing and knowledge of the intervention on outcome were controlled and validity of outcome measures.

A generally descriptive analysis was selected as most appropriate for the research question, because of the heterogeneity of the studies that were identified. However, a meta-analysis was also carried out involving published and unpublished results from five studies which looked specifically at body/support interface pressure under the ischial tuberosities. A more conservative random effect model was used rather than a fixed effect model due to the presence of heterogeneity across the studies.^14^

It was not possible to directly combine all the data in one meta-analysis as two of the studies^15^,^16^ had reported measurements taken from the same participants while sitting on different cushion configurations and therefore the data sets were not considered truly independent of one another. However two separate meta-analyses were carried out, using the results for specific cushions in each study corresponding to the best and worse case scenarios (i.e. most and least pressure reduction).

## Results

Of the 389 publications identified in the electronics database searches, only 15 fulfilled the selection criteria (Appendix 1). An additional five publications were identified by other means. Two publications referred to the same study.

Nineteen studies were identified (Table 2). All of the studies were on populations with neurological impairment. Ten of the studies were on young people: with cerebral palsy (*n* = 8), neural tube defect (*n* = 1), or unspecified neurological impairment (*n* = 1). Nine of the studies were on adults: with spinal cord injury (*n* = 8) or multiple sclerosis (*n* = 1).

**Table 2:** Summary of methods

Ref.	Study design	Participants	Type of seat	Tilt angles	Timing and order of tilt	Comments	Evidence level
(a) Studies involving posterior tilt							
3	Case study	1, 9-year old with CP	Wheelchair including head rest, lateral and anterior trunk support, foot support. 90° seat-to-back angle	0°, 15° and 30° posterior tilt	10 sessions with 20 min at each tilt angle in variable order		5
4	1: Cross-over study	10 adults with MS	Same manual wheelchair	0° and 25° posterior tilt	15 min upright then 15 min tilted		4
	2: RCT	20 adults with MS		0° and 25° or 45° posterior tilt	Upright then tilted. No acclimatization period	Subjects randomly assigned to 25° or 45° tilt angle	2
5, 20	Cross-over study	12 adults with complete SCI	Reclining/tilting wheelchair with seat cushion, arm and foot rests. 100° seat-to-back angle	0°, 10° and 20° posterior tilt	Set order for testing positions. 15 min acclimatization in each position	6 other positions tested within session	4
6	Cross-over study	2 adults with C5 quadriplegia	Subjects’ own wheelchairs with 100° seat-to-back angle	0°, 35° and 45° posterior tilt	Single session with set order for positions: 0°, 35°, 45° and 0° (repeated)	2 other positions tested. Repeated on 3 seat cushions	5
7	Cross-over study	15 children (7–18 years) with myelo- meningocele	Chair with back and head rest, foam cushion on base, 90° seat-to-back angle	0° and 25° posterior tilt	Randomized order for positions with each position repeated twice. 30 seconds data collection in each position	3 other positions also tested	2
15	Cross-over study	16 adults, SCI, motor complete tetraplegia	Subjects’ own powered wheelchairs. 95° median seat-to-back angle	5° median and 45° posterior tilt	Upright for 1 min, then tilted for 1 min	Repeated on 2 seat cushions. Inter-subject position variations	4
16	Cross-over study	18 adults with complete SCI (C5–L2)	Powered wheelchair with 90° seat-to-back angle	5°, 15° and 25° posterior tilt	3 sessions with all conditions tested in random order in session. 15 seconds in each condition	Testing repeated at 3 cushion inflation pressures	2
17	Cross-over study	11 children (4–8 years) with spastic CP	90° seat-to-back angle, head rest, lateral trunk supports, adductor wedge; foot rest	0° and 150 posterior tilt	Single session with random order to positions. 3 min acclimatization in each position	5 other positions also tested	2
19	Cross-over study	20 adults with complete thoracic SCI	Chair with back and foot rests, 100° seat-to-back angle	0°, 7° and 12° posterior tilt	Single session with random tilt order. Each position repeated twice	1 other position tested. Subjects undertook reaching task during measurement	2
21	Cross-over study	14 adults, C6-T10 motor complete SCI	E&J Premier (upright) and Quickie Breezy 500 (4° posterior tilt) wheelchairs. 90° seat-to-back angle	0° and 4° posterior tilt	Single session, with random order for testing chairs. Time in chairs not specified	Additional differences between wheelchairs. Chair with acute seat-to-back angle also tested	4
25	Cross-over study	10 adults with SCI	Subjects’ own wheelchair with seat cushion	0°, 35° and 65° posterior tilt	Single session with set order for positions: 0°, 35°, 65°	Another position also tested	4
27	Cross-over study	12 children (6–18 years) with CP (spastic diplegia)	As in ref. 18. Hip abductor also included	0° and 30° posterior tilt	Single session with random tilt order. 5 min acclimatization in each position		2
29	Cross-over study	6 children with spastic CP, mean age 6 years	Upholstered seat base, foot rests. No back rest nor arm rests	0° and 10° anterior tilt	2–3 min acclimatization, 5 min upright 5 min tilted. Repeated at 3 sessions	Subjects independent sitters and ambulatory	4
(b) Studies involving anterior and posterior tilt							
22	Case series	23 children with CP (2–16 years)	Range of seats, providing foot, pelvic and trunk support. Seat-to-back angle ranging from 9° to 130°	0–30° posterior (mean 8°) and 0–15° anterior tilt (mean 8°)	Single session with random order to positions and 5 min in each position	Tilt angles varied between subjects. Seats also varied between subjects and tilts. Repeated with table and abductor	5
24	Cross-over study.	10 children with spastic CP	90° seat-to-back angle, head rest, lateral trunk supports, chest panel, foot rest	0°, 15°, 30° posterior and 15° anterior tilt	Random order to tilt positions, then repeated in reverse order. 5 min acclimatization in each position	3 children with athetoid CP also measured (analysed separately)	2
28	Cross-over study	10 children with spastic CP (4–15 years)	Chair with back rest and foot support. 90° seat-to-back angle changed to 95° for anterior tilt	0°, 5° posterior and 5° anterior tilt	3 sessions with one randomly selected tilt angle per session	Measurements during ‘quiet sitting’ and during upper extremity activity	2
(c) Studies involving anterior tilt							
18	Cross-over study	15 children (2–6 years) with developmental delay and/or CP	Adjustable bench with non-skid surface	0°, 20° and 30° anterior tilt	Single session of 30 min. Random order for testing positions with 1 min acclimatization in each position	2 other positions/seats measured. Limited control over bench postures	4
26	Cross-over study	20 adults with complete thoracic SCI	Chair with flat/ramped seat base, foot support, and support behind trunk.	0° and 10° anterior tilt	Single session. Upright then tilted	Base only tilted. Subjects undertook reaching task	4
23	Cross-over study	14 children with CP (5–11 years)	Upholstered seat base, foot rests. No back rest nor arm rests	0°, 10° and 15° anterior tilt	Four 20-minute sessions each at 2 tilt angles (0–10°, 10–0°, 0–15°, 15–0°)	Subjects independent sitters and ambulatory	2

CP, cerebral palsy; MS, multiple sclerosis; SCI, spinal cord injury.

The seat was tilted anteriorly by up to 30° in three of the studies, was tilted posteriorly by up to 45° in 13 studies and was tilted in both directions in three studies.

Several studies included additional interventions. Additional seat configurations and postures were included in the studies of Nwaobi *et al*.^17^ Miedaner,^18^ Pellow,^6^ Vaisbuch *et al*.,^7^ Janssen-Potten *et al*.^19^ and Hobson.^5^,^20^ The seat cushions also varied in the studies of Burns and Betz^15^ and Spijkerman *et al*.,^16^ who examined effects on interface loading. Hastings *et al*.^21^ compared three designs of wheelchair, two of which had different, fixed tilt angles. Myhr and von Wendt^22^ compared postures in individuals’ own seats with postures in an alternative seat which was adjusted to provide a more forward-inclined position.

Seventeen of the studies were essentially cross-over trials comparing seat orientation (Table 2). Myhr and von Wendt's study^22^ can be considered as a series of case reports because of the range of seats and orientations involved in the intervention. In another study^3^ a single child was seated at three angles of tilt. In the second part of Chan and Heck's study^4^ subjects were randomly assigned to two groups that were tilted back to two different angles of tilt.

In 10 studies the order of tilt was randomized at each measurement session. Two studies^23^,^24^ looked at ordering effects by repeating the measurements in a reverse order and comparing outcomes. In one study the full set of seat positions were measured over multiple sessions^23^. In the other studies all seat positions seemed to be measured in a single session. The measurement period in each position varied between a few seconds to 20 minutes. It was not possible to blind the subject or the researcher to the intervention(s) in any of the studies.

Outcomes included: interface pressure,^5–7^,^15^,^16^,^20^,^25^ shear force,^5^,^20^ surface EMG,^17^,^19^,^23^,^26^,^27^ postural measurements,^4^,^18^,^21^,^23^,^26^ change in head position^23^,^28^, timed upper extremity activity,^24^,^28^ respiratory measurements,^4^,^29^ voice volume^4^ and perceived exertion^4^ (Table 3).

**Table 3:** Evidence of effect in studies: outcomes measures

Ref.	Outcome measure	Tilt away from vertical	Mean change with tilt from vertical	Significance level reported (*P* = 0.05)
Interface loading				
5, 20	Maximum pressure under ischial tuberosities	20° posterior tilt	−11%	Yes [in ref. 20]
	Tangential shear force through seat		−85%	Yes [in ref. 20]
6	Pressure at ischial tuberosities and sacrum (averaged over the 3 locations, mean over 1 minute of measurements)	45° posterior tilt	−34%	Not reported (2 participants)
7	Maximum interface pressure	25° posterior tilt	−22%	Yes (*P* < 0.01)
	Mean interface pressure (mean of 2 measurements)		−8%	No
15	Pressure under ischial tuberosity (side of highest pressure, mean of 10 measurements)	45° posterior tilt	−33%	Yes (*P* < 0.001)
16	Maximum pressure under right ischial tuberosity; average for 3 cushion inflation pressures, 3 measurements at each	20° posterior tilt	−5%	Yes (*P* = 0.012)
25	Pressure over ischial tuberosities, mean over 1 minute of measurements	35° posterior tilt	−27%	No
Posture and stability				
4	Thoraco-lumbar distance	25° posterior tilt	+3%	No
	Cervico-thoracic distance		−36%	Yes
21	Thigh length (indirect measure of pelvic tilt), shoulder position and head orientation from photographs	14° posterior tilt	−1.1−1.6 cm, +6.5°, respectively	No
28	Mean displacement of the head,^#^ shoulder,^#^ hip knee, ankle	5° posterior tilt, 5° anterior tilt	Variable. Maximum change was 4 cm increase	Yes^#^ (in some segments with anterior tilt)
26	Sagittal pelvic orientation	10° anterior tilt	<2° more anterior. Variable	No
18	Distance from pelvis to spinous process	30° anterior tilt	−8%	Yes
23	Sitting height	15° anterior tilt	−0.21 cm	No
	Radius of head position (stability)		−0.97 cm	Yes (*P* = 0.037)
Muscle activity				
17	EMG (lumbar erector spinae)	15° posterior tilt	+ 37%	No
19	EMG (erector spinae at T3, T9 and L3, serratus anterior,^†^ oblique abdominals,^†^ pectoralis major,^†^ latissumus dorsi,^†^ trapezius)	12° posterior tilt	Variable. Increased in some groups. Decrease in others	Yes^†^ in some muscles and injury levels, no for others
27	EMG (iliocostalis lumborum, adductor magnus and gastrocnemius)	30° posterior tilt	+51, +19, +1%, respectively	Yes (back and hips)
23	EMG (erector spinae, average from four bilateral paraspinal sites)	15° anterior tilt	+73%	Not reported
26	EMG (erector spinae at T3,* T9* and L3,* oblique abdominals, serratus anterior, pectoralis major, latissumus dorsi, trapezius*)	10° anterior tilt	Up to −50% depending on anatomical location and level of injury	Yes* in some muscles and injury levels, no for others
Respiratory function				
4	Forced vital capacity	25° posterior tilt	+20%	Yes (*P* < 0.001)
	Chest expansion		+7%	Yes (*P* = 0.014)
29	Tidal volume, respiration rate, minute ventilation	10° anterior tilt	+12, +3, +3%, respectively	No
Other functional activity				
19	Maximum unsupported forward reach distance	12° posterior tilt	<5 cm difference	No
3	Time with head directed to activity	15° posterior tilt	+22%	Not reported
4	Voice volume	25° posterior tilt	−0.1%	No
	Perceived exertion on Borg's scale of 6–20		−4.96%	No
24	Timed switch use with upper extremity	30° posterior 15° anterior tilt	+39%, +44%, respectively	Yes Yes
28	Upper extremity activity (6 timed tasks)	5° posterior 5° anterior tilt	Improved in 1 of 6 tasks in each tilt condition	Yes for only 1 task No for 5 tasks.
22	Time with head upright	0–15° anterior tilt (mean 8°)	+93% mean duration	Yes (*P* = 0.001)
	Sitting assessment score 5–20		+56% median score	Yes (*P* = 0.001)
	Number of pathological movements		−75% median number	Yes (*P* = 0.002)

### Meta-analysis of interface pressure

**Figure 2:**
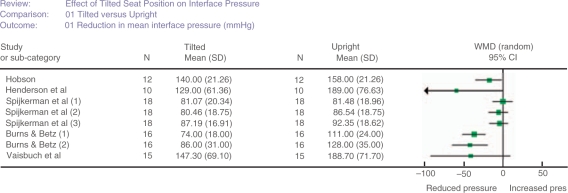
Forest plot showing results of studies investigating body/support interface pressure under the ischial tuberosities. The participants in the studies by Hobson^5^,^20^ and Vaisbuch *et al*.^7^ were sitting on foam seat cushions while the participants in the study by Henderson *et al*.^25^ remained sitting on their own personal cushions. Spijkerman *et al*.^16^ used dry flotation seat cushions and repeated measurements with the same participants sitting on cushions inflated to 20 mmHg (1), 30 mmHg (2) and 40 mmHg (3). Burns and Betz^15^ repeated measurements with participants sitting on a dry flotation seat cushion (1) and a gel seat cushion (2).

Figure 2 shows a forest plot for five of the six studies that investigated interface pressure under the ischial tuberosities.^5^,^7^,^15^,^16^,^25^ It was not possible to include Pellow's study^6^ as insufficient data were reported and there were only two participants. Spijkerman's^16^ unpublished data were used in the analysis and it was necessary to make a conservative calculation of the standard deviation from the reported significance level for Hobson's study.^5^,^20^ Multiple results shown for particular studies^15^,^16^ relate to the use of different seat cushions. An inspection of Figure 2 suggests a reduction in interface pressure when participants were posterior tilted (between 20° and 45°) compared with upright.

The worst case scenario in terms of pressure reduction suggested a reduction of 24.00 (95% confidence interval (CI) 4.19–43.80) mmHg (*P* = 0.02). The best case scenario was a reduction of 24.80 (95% CI 7.16–42.44) mmHg (*P* = 0.006).

## Discussion

The restriction of the search to papers written in English may have limited the findings of the review. The comparative difficulty in identifying unpublished studies compared to published work may also have limited the findings.

Wide search criteria were used in the systematic review because there was not thought to be much evidence available on the effects of tilted positions. Therefore selection included studies on a range of populations, interventions, experimental methodologies and outcomes.

Studies on different populations (spinal cord injury and neural tube defect) and at different tilt angles were included in the meta-analysis. There were insufficient data to rigorously test the validity of this strategy.

Studies included in the meta-analysis were randomized^7^,^16^ and non-randomized^5^,^15^,^25^ trials where participants acted as their own controls. Ideally the order of tilt orientation should have been randomized in all the studies that were included in the meta-analysis as this would remove a potential source of bias.

### Experimental design

Most of the studies were cross-over trials looking at the immediate effects of seat orientation on the seated person. With a cross-over experimental design there is potential for tilt order to affect outcome due to fatigue and other physiological responses. Tilt order will also affect outcome if there are changes to the baseline sitting posture during the experimental procedure due to sliding in the seat. In a cross-over study it is feasible to control the effects of order of tilt through experimental design. Measurement at different tilt angles may take place in different sessions. Alternatively, the tilt sequence may be randomized across the sample or the measurements at each tilt angle may be repeated in a different order. These approaches are recommended in future cross-over studies.

It is also possible for knowledge of the seat orientation during the experimental protocol to affect the outcome. Unfortunately it is not practical to blind the subject or experimenter to the orientation of the seat.

The quantitative studies which compared outcomes on different seats (or at different tilt angles) involved small samples of fewer than 20 people. There is potential for actual differences between tilt angles or seats not to be identified as significant because of the distribution of data within the small samples (a type II error). As the number of reported results increase, there will be scope for additional meta-analysis.

In some of the studies on the effects of an anterior seat tilt the intervention comprised a forward tilt of the seat base without additional support about the pelvis or trunk.^18^,^23^,^26^ The variation in findings between studies may be because protocols did not control for other influences on posture.

The number of seating systems on the market providing an anterior tilt is limited, and such seating is not widely used. This may be due to difficulties using these systems in vehicle transport and using them with desks and powered mobility systems. For this reason an investigation into the effects of forward tilt may not be the highest priority for the next stage of research.

No cohort studies were identified which investigated longer term effects of tilt-in-space usage with a quantitative methodology. This approach may be worth considering for future work.

### Outcomes

Outcomes measures in most of the studies were related to abnormality of anatomical structure or function (impairment). There was little consideration of the importance of any differences that were identified to the health or social participation of the user.

Six studies reported that tilting the seat back reduced the pressure under the ischial tuberosities in a range of conditions. However the sample sizes involved in the above studies were relatively small and the methods of statistical analysis and levels of significance (when reported) varied noticeably. Pooling data across five of these studies in a meta-analysis produced more robust evidence of a statistically significant reduction in pressure under the ischial tuberosities when participants are tilted backward compared to when upright.

Hobson's^5^,^20^ finding of reduced frictional shear stress underneath the seat base with a 200 posterior tilt, is consistent with a generalized biomechanical analysis of a seated person.^30^

Loading at the interface with the seat is likely to influence susceptibility to pressure ulcers and comfort during sitting. A cohort study on pressure ulcer prevalence in tilt-in-space wheelchair users compared with in a control group of conventional wheelchair users would identify whether the reduction in loading when tilted backwards results in reduced pressure ulcer prevalence for tilt-in-space users.

Studies in different muscle groups and in the cerebral palsy and spinal cord-injured populations have reported that EMG activity in some muscle groups is affected by tilt.^19^,^26^,^27^ In populations and muscle groups where raised activity restricts functional movements and leads to the development of contractures, decreased activity may be advantageous. Reduced muscle activity may also be associated with reduced effort during movements or with the maintenance of position. However in other populations and circumstances, increased muscle activity may be associated with increased functional movements and improved posture. Overall, the effect of seat tilt on EMG activity and how that affects functional outcomes has not been established.

Postural measurements were either between anatomical markers, or between an anatomical marker and the seat surface and were focused on trunk and head position in the sagittal plane. In the studies where measurements were taken from photographs or video frames^21^,^28^ there was potential for error from neglected out-of-plane components of position. The postural results overall were inconclusive (Table 3).

Head control has been assessed from measurements^23^,^28^ of head position over time. Sochaniwskyj^23^ used a potentiometric linkage, however the measurements were not set into a functional context. Head control has also been assessed from observations of head position over time,^3^,^22^ but in Myhr and von Wendt's study^22^ the inter-rater reliability of the observers was reported for only two of six positions and ranged from 0.9 to 0.31 using Spearman's rank correlation coefficient.

Ability to perform an activity from the seat is a key aspect of any study on the effects of seat tilt. Nwaobi^24^ used timed switch operation and McClenaghan *et al*.^28^ used timed tasks as measures of upper extremity function. Myhr and von Wendt ^22^ evaluated hand and arm function using observational techniques and a rating scale. Respiratory measurements were included as an outcome in only two of the studies identified by this review. Reid and Sochaniwsky^29^ made indirect measurements of tidal volume via plethysmography whereas Chan and Heck^4^ took measurements of vital capacity using lung function spirometry. Additional studies on capabilities in tilted postures for specific populations would be worth while.

No studies on ability to transfer into and out of the seat were identified in the populations of interest. Studies on other more ambulatory populations^31^ have suggested that ability to independently transfer may be reduced by a posteriorly tilted position. However many people within the populations that are covered by this review have to use a hoist to transfer into and out of the seat, so the effect should be investigated separately.

### Effects within populations

No studies were identified on the effects of seat tilt on people with progressive neuromuscular conditions (e.g. muscular dystrophy). This population would benefit from study, as the question of whether to provide a tilt facility on a wheelchair is a common clinical issue.

The studies with cerebral palsy were on young people and tended to measure posture and muscle activity. However it was not possible to identify consistent finding from these studies due to variation in interventions, outcome measures and heterogeneity of the population. The use of the Gross Motor Function Classification System^32^ in future investigations to identify the participants’ level of physical ability would enable clinicians to judge the advantages and disadvantages of varying angles of tilt for specific children.

Most of the studies in populations with spinal cord injury and neural tube defect were on the effects of seat tilt on interface loading. This is an important outcome in populations that are prone to pressure ulcers.

The only quantitative study that was identified was one on people with multiple sclerosis by Chan and Heck.^4^ Themes which emerged from in-depth interviews^2^ with this population included prolonged sitting up during day and fatigue. Chan and Heck^4^ attempted to identify the immediate effects of a change in orientation on fatigue using Borg's Rating of Perceived Exertion scale. However, the increase in fatigue with tilt that was identified is likely to be affected by their protocol, which involved a fixed order of tilt.

Previous cohort studies on how fatigue, duration of sitting, other health and social factors are affected by long-term use of a tilted position have not been identified. Future cohort studies on tilt-in-space seat usage, compared with standard seat usage would greatly inform clinical practice.

**Clinical messages**

- Evidence is lacking on the effects of tilted seat positions on health, function and participation outcomes.
- Studies on progressive neurological/neuromuscular populations are particularly scarce.
- There is some evidence to suggest a posterior seat tilt reduces pressures under the pelvis for people with neurological impairment.

## Conclusions

Results from studies on populations with spinal cord injury and neural tube defect suggest that a posterior seat tilt of 20° or more reduces pressures under the pelvis.

Overall there is a lack of quality evidence to support and guide the use of the tilted position in seating for populations with neurological and neuromuscular impairment. Current evidence is weakened by mixed interventions and confounding factors. Outcome measures, participants and interventions need to be determined more rigorously to ensure that confounders do not reduce the quality and usefulness of future studies.

A priority area for future studies might be effect of posterior seat tilt on functional activity and seat use, in populations with progressive neuromuscular conditions.

### Competing interests

None declared.

## Appendix 1 Literature search: main terms and publications identified

**Table:**

		Total found	New publications selected	Duplicates
1	Wheelchairs/and tilt$.mp (MEDLINE, CINAL, AMED)	57	9	0
2	Wheelchair/and tilt$.mp (Embase)	42	1	8
3	Seat/and tilt$.mp (Embase)	31	2	4
4	Seating/and tilt$.mp (CINAL, AMED)	28	0	6
5	Sitting/and tilt$.mp (Embase, CINAL, AMED)	116	0	5
6	Wheelchairs/and tip$.mp, (MEDLINE, CINAL, AMED	39	2	0
7	Wheelchair/and tip$.mp (Embase)	30	0	1
8	Seat/and tip$.mp (Embase)	6	0	1
9	Seating/and tip$.mp, (CINAL, AMED)	4	1	1
10	Sitting/and tip$.mp (Embase, CINAL, AMED)	36	0	3
	Hand search of reference lists, consultation with experts in the field	–	5	–
